# Why Has the Ability to Regenerate Following CNS Injury Been Repeatedly Lost Over the Course of Evolution?

**DOI:** 10.3389/fnins.2022.831062

**Published:** 2022-02-04

**Authors:** Seth Blackshaw

**Affiliations:** ^1^The Solomon H. Snyder Department of Neuroscience, Johns Hopkins University School of Medicine, Baltimore, MD, United States; ^2^Department of Ophthalmology, Johns Hopkins University School of Medicine, Baltimore, MD, United States; ^3^Department of Neurology, Johns Hopkins University School of Medicine, Baltimore, MD, United States; ^4^Institute for Cell Engineering, Johns Hopkins University School of Medicine, Baltimore, MD, United States; ^5^Kavli Neuroscience Discovery Institute, Johns Hopkins University School of Medicine, Baltimore, MD, United States

**Keywords:** regeneration, neurogenesis, vertebrate, mammal, zebrafish, reactive gliosis, infectous disease, axon

## Abstract

While many vertebrates can regenerate both damaged neurons and severed axons in the central nervous system (CNS) following injury, others, including all birds and mammals, have lost this ability for reasons that are still unclear. The repeated evolutionary loss of regenerative competence seems counterintuitive, and any explanation must account for the fact that regenerative competence is lost in both cold-blooded and all warm-blooded clades, that both injury-induced neurogenesis and axonal regeneration tend to be lost in tandem, and that mammals have evolved dedicated gene regulatory networks to inhibit injury-induced glia-to-neuron reprogramming. Here, different hypotheses that have been proposed to account for evolutionary loss of regenerative competence are discussed in the light of new insights obtained into molecular mechanisms that control regeneration in the central nervous system. These include pleiotropic effects of continuous growth, enhanced thyroid hormone signaling, prevention of neoplasia, and improved memory consolidation. Recent evidence suggests that the most compelling hypothesis, however, may be selection for greater resistance to the spread of intra-CNS infections, which has led to both enhanced reactive gliosis and a loss of injury-induced neurogenesis and axonal regeneration. Means of testing these hypotheses, and additional data that are urgently needed to better understand the evolutionary pressures and mechanisms driving loss of regenerative competence, are also discussed.

## The Evolutionary Loss of Injury-Induced Regenerative Competence in Central Nervous System

Most fish and amphibian species can efficiently regenerate damaged neurons and axons in the brain, spinal cord, and/or retina following injury. Regeneration can be robust, rapid, and reproducible: zebrafish, for instance, are able to completely replace retinal photoreceptors lost following light damage more than six times without any clear behavioral defects or morphological abnormalities (Ranski et al., 2018). Neuronal regeneration typically occurs by dedifferentiation of glial cells into neuronal progenitors, which then generate new neurons (Tsonis and Del Rio-Tsonis, 2004; Lust and Tanaka, 2019). In salamanders, neuronal regeneration can also occur through dedifferentiation of ependymal cells or transdifferentiation of retinal pigment epithelium (Lust and Tanaka, 2019).

While regenerative competence likely represents an ancestral state for the vast majority of vertebrate species, injury-induced neurogenesis and axonal regeneration have been repeatedly and independently reduced or lost over the course of evolution. For instance, regenerative competence is greatly reduced or lost entirely in the brain and retina of certain fish and amphibians, including medaka (Lust and Wittbrodt, 2018; Shimizu and Kawasaki, 2021) and the clawed frog *Xenopus tropicalis* (Langhe et al., 2017). Since regenerative competence has been carefully assayed in only a small number of species (Lust and Tanaka, 2019), this is likely to also be true for many other teleost and amphibian species. Furthermore, regenerative competence is far more limited in amniotes. Injury-induced regeneration in adult reptiles is restricted to a few CNS regions, including the forebrain and the tail spinal cord (Duffy et al., 1990; Font et al., 2001). Injury-induced neurogenesis is observed in juvenile (but not adult) chick retina (Fischer and Reh, 2001), and also in the forebrain of adult ring doves, although mammals essentially lack regenerative competence altogether. Regenerative competence in the CNS usually declines with age, sometimes dramatically so (Tanaka and Ferretti, 2009; Yun, 2015; Alunni and Bally-Cuif, 2016). Finally, injury-induced regeneration in the CNS is often but not always correlated with competence to regenerate non-neuronal tissues such as cardiac muscle (Tanaka and Ferretti, 2009; Alunni and Bally-Cuif, 2016; Alesci et al., 2021).

This evolutionary loss of regenerative competence is puzzling. Why would such a seemingly useful ability be independently discarded in multiple evolutionary lineages? Is regeneration following CNS injury actually disadvantageous for many species, or does this reflect an unavoidable trade-off for some other beneficial trait, and if so, what? There are several facts that need to be accounted for in any explanation of this phenomenon. First, while regenerative competence seems to be an evolutionary basal state, it has been lost in many species (Alunni and Bally-Cuif, 2016). Second, loss of competence to regenerate both destroyed neurons and severed axons usually go hand in hand (Ferretti and Géraudie, 1998; Curcio and Bradke, 2018). Third, while glial cells in regenerative species transition rapidly through a reactive-like state before reprogramming into neurogenic progenitors, glial cells in regeneration-incompetent clades such as mammals instead arrest in this state, massively prolonging the duration of reactive gliosis, and often also showing scarring and fibrosis (Burda and Sofroniew, 2014; Escartin et al., 2021). Lastly, recent work (Hoang et al., 2020) shows that dedicated gene regulatory networks inhibit injury-induced glial reprogramming. Here, I critically evaluate explanations that have been proposed to account for the evolutionarily loss of regenerative competence in the light of these observations, discuss experimental approaches to investigate these hypotheses, and highlight other unresolved questions in the field.

## Proposed Explanations for Evolutionary Loss of Central Nervous System Regenerative Competence

### Hypothesis 1: Regenerative Competence Is Lost as the Indirect Result of Loss of Continuous Tissue Growth

Both the body and brain of most cold-blooded vertebrates continue to grow throughout life, and add neurons at all points along the neuraxis to compensate. This has led to the proposal that regenerative competence in adult CNS both arises, and is indirectly lost, in response to adaptations that allow or prevent continuous tissue growth, and is dependent on the presence of dedicated neuronal progenitors that remain active in the CNS throughout life (Holder and Clarke, 1988; Tanaka and Ferretti, 2009). This model makes several testable predictions. First, regenerative competence should correlate directly with ongoing adult neurogenesis. Second, newly generated neurons should typically arise from the same resident neuronal progenitors that contribute to constitutive neurogenesis.

In many cases, patterns of injury-induced neurogenesis do indeed correlate closely with patterns of constitutive neurogenesis, such as in the forebrain of non-mammalian vertebrates (Tanaka and Ferretti, 2009; Grandel and Brand, 2013; Alesci et al., 2021). Injury-induced neurogenesis almost invariably occurs in species and/or CNS regions where continuous neurogenesis is present, implying that ongoing constitutive neurogenesis may be necessary for injury-induced neurogenesis to occur. Furthermore, in some cases, neurons generated as the result of continuous and injury-induced neurogenesis clearly arise from the same progenitor cell population. This is dramatically illustrated by the example of the zebrafish cerebellum, where granule cells are generated throughout life by fate-restricted progenitors, but are the only neuronal cell type regenerated following injury in adulthood (Kaslin et al., 2013, 2017).

In other cases, however, the correlation between ongoing adult neurogenesis and regenerative competence is not precise, with injury-induced neurogenesis often absent where ongoing neurogenesis is present. For instance, the teleost medaka, which shows only highly limited regenerative competence, displays both continuous growth and ongoing neurogenesis (Lust and Wittbrodt, 2018; Shimizu and Kawasaki, 2021). Conversely, while a few discrete mouse brain regions, such as the hippocampal dentate gyrus, show ongoing neurogenesis well into adulthood (Ming and Song, 2011), cells in this region show only limited ability to regenerate damaged neurons following injury, and no evidence for either injury-induced reprogramming of glia or axonal regeneration (Alunni and Bally-Cuif, 2016). These imply that regenerative competence is not necessarily correlated with levels or patterns of ongoing adult neurogenesis.

Furthermore, neurons generated in response to injury can in some cases arise from different progenitor populations than those used in ongoing adult neurogenesis. The teleost retina contains two populations of resident neurogenic neural progenitors: the ciliary margin zone at the retinal periphery which can generate any neuronal subtype, and a radial glial-like population found throughout the retina that is limited to generating rods (Stenkamp, 2007). Injury-induced neurogenesis, however, is generally thought to occur through reprogramming of a third population—quiescent resident Müller glial cells—to a neural progenitor-like state (Gorsuch and Hyde, 2014; Wan and Goldman, 2016; Lahne et al., 2020), although there remains some controversy as to whether rod-restricted progenitors are truly distinct from the broader population of Müller glia (Stenkamp, 2011).

Lastly, this model does not account for the loss of competence to regenerate severed axons, the extended period of reactive gliosis seen following injury in regeneration-incompetent species, or the active repression of neurogenic competence seen in mammals. On balance, the loss of regenerative competence is highly unlikely to simply occur as an indirect result of the loss of continuous neurogenesis, although this may be so in some cases. Directly testing the model would require the ability to selectively disrupt ongoing neurogenesis without also affecting competence for injury-induced neurogenesis. Since both constitutive and injury-induced neurogenesis are likely to use many of the same molecular mechanisms, both cell-specific and conditional genetic approaches will be needed to address this (Hans et al., 2021).

### Hypothesis 2: Regenerative Competence Is Lost as the Result of Increased Thyroid Hormone Signaling

Thyroid hormone levels are often inversely correlated with regenerative competence. Thyroid hormone signaling actively inhibits cardiac muscle regeneration (Hirose et al., 2019; Marshall et al., 2019). Thyroid hormone acts as a trigger for amphibian metamorphosis (Brown and Cai, 2007), following which regenerative competence is substantially reduced. Increased thyroid hormone levels also accelerate developmental loss of axonal regeneration in cerebellar Purkinje cells (Avci et al., 2012). This has led to the proposal that CNS regenerative competence may have been indirectly suppressed as the result of selection for increased thyroid hormone signaling (Tanaka and Ferretti, 2009). This model predicts that thyroid hormone signaling will actively inhibit regenerative competence in all vertebrates, and that its disruption should restore regenerative competence in regeneration-incompetent species such as mammals.

Evidence to support this model is limited, however. Although regenerative competence is decreased relative to the larval stage, salamanders and *Xenopus laevis* retain regenerative competence following metamorphosis (Alunni and Bally-Cuif, 2016; Alesci et al., 2021). Furthermore, thyroid hormone signaling stimulates both adult neurogenesis and functional recovery following injury (Montero-Pedrazuela et al., 2006; Fanibunda et al., 2018). Finally, the effects of manipulating thyroid hormone signaling in regeneration-competent zebrafish are complex. Both dose and context-dependence are observed, with thyroid hormone in some cases stimulating regeneration (Bhumika and Darras, 2014). While thyroid hormone signaling may indeed modulate regenerative competence, its effect is not consistently positive or negative. Nonetheless, regenerative competence has not been carefully tested in either dietary-induced hypothyroidism, nor following genetic disruption of either thyroid hormone synthesis or signaling. It remains formally possible that reductions in thyroid hormone signaling may restore some levels of regenerative competence to mammals.

### Hypothesis 3: Loss of Regenerative Competence Is an Adaptation That Promotes Resistance to Cancer

High levels of ongoing cell proliferation are associated with increased mutation rates and formation of cancers. Since a reduction in proliferation may indirectly inhibit neoplasia, this has been proposed as a mechanism that underlies the loss of regenerative competence in longer-lived species (Tanaka and Ferretti, 2009; Chung, 2018). This model predicts that regeneration-competent species will show higher rates of cancers such as glioblastoma and neuroblastoma-like tumors. In addition, regenerative competence might be expected to decrease with age, as the probability of tumor formation increases (Kitsoulis et al., 2020).

It is difficult to evaluate the accuracy of this hypothesis, as tumor incidence in regeneration-competent vertebrates has generally not been extensively characterized. However, tumor incidence in zebrafish is lower than in mice for both spontaneous and genetically induced tumors (Feitsma and Cuppen, 2008; Hason and Bartůněk, 2019). Furthermore, while tumor incidence rises with age in zebrafish as in mammals (Raby et al., 2020), no clear age-dependent reduction in regenerative competence is observed (Yun, 2015). Cancer rates are also highly variable among regeneration-incompetent mammalian species, and are inversely correlated with reproductive lifespan (Albuquerque et al., 2018). It does not explain why axonal regeneration would be lost along with injury-induced neurogenesis, or why gliosis is extended in regeneration-incompetent species. Overall, this does not appear to be a likely explanation for the evolutionary loss of regenerative competence.

### Hypothesis 4: Loss of Regenerative Competence Promotes Improved Memory Consolidation

Regeneration-induced remodeling of the CNS can, in principle, disrupt pre-existing patterns of neuronal connectivity and synaptic potentiation. In light of the loss of regenerative competence in mammals, it has been proposed that this loss has occurred secondarily to enhanced long-term memory (Kiernan, 1979; Larner et al., 1995). This model predicts that cold-blooded vertebrates with reduced regenerative competence, such as medaka, should show improved long-term memory. Furthermore, neurogenesis and axonal regeneration should disrupt long-term memory consolidation.

There is currently no concrete evidence to support this hypothesis, although long-term memory retention following neuronal regeneration has yet to be investigated. Adult neurogenesis in the olfactory bulb and dentate gyrus in mammals has been linked to long-term memory formation, not erasure (Gage, 2019; Tuncdemir et al., 2019). Loss of regenerative competence occurs in both the brain and other CNS regions, such as retina and spinal cord, where experience-dependent plasticity is substantially lower (Tanaka and Ferretti, 2009; Alunni and Bally-Cuif, 2016; Alesci et al., 2021). Likewise, there is no evidence that regeneration-incompetent species show improved long-term memory, although this awaits rigorous investigation. Overall, this too seems an unlikely explanation for loss of regenerative competence.

### Hypothesis 5: Loss of Regenerative Competence Increases Resistance to Infection

Infectious diseases and parasites are a common cause of premature mortality in virtually all species, and resistance to infection is a major selective pressure (Karlsson et al., 2014). Since the CNS is largely isolated from ongoing adaptive immune surveillance (Forrester et al., 2018), the consequences of breakthrough infections are potentially severe. A final hypothesis to explain the evolutionary loss of regenerative competence is that it confers resistance to intra-CNS infection, both by blocking proliferation and axonal outgrowth of infected cells and by enhancing and prolonging reactive gliosis (Rattner and Nathans, 2006; Hoang et al., 2020).

**FIGURE 1:**
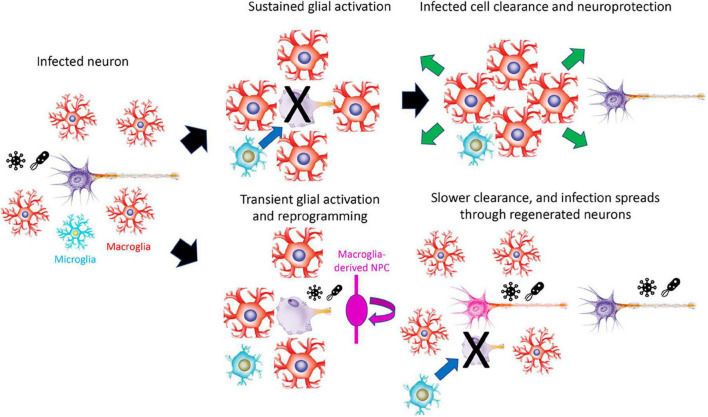
Model for how prolonged reactive gliosis and loss of regenerative competence may protect the CNS against infection. Infection leads to extended reactive gliosis and destruction of infected cells in regeneration-incompetent organisms, restricting the spread of infection. In regeneration-competent organisms, however, limited gliosis and rapid regeneration can lead to intra-CNS spread of infection. For clarity, macroglia are shown as astroglial-like rather than the radial morphology adopted in many regeneration-competent species. NPC, neural progenitor cell.

In contrast to the other models proposed, this largely accounts for the observed differences in injury response seen between regeneration-competent and -incompetent organisms. It explains the otherwise mysterious phenomenon of the enhanced and prolonged reactive gliosis observed following injury in mammals (Burda and Sofroniew, 2014). Reactive gliosis consists of three major components. Activated glia first swell and become rigid, forming a physical barrier. Working in tandem with microglia, they then kill and consume damaged cells, and in parallel, they release a cocktail of secreted factors that protect cells not targeted for destruction. Genetic studies have demonstrated that reactive gliosis greatly restricts the spread of intra-CNS pathogens in non-regeneration competent species (Drögemüller et al., 2008; Burda and Sofroniew, 2014). Enhancing and prolonging this process, while also eliminating pathogen spread that is potentially induced by proliferation, neurogenesis and axonal outgrowth, is a potent defense against the spread of infection (Figure 1). While modest levels of inflammation are required to initiate glial activation and reprogramming (Kyritsis et al., 2012; Wan and Goldman, 2016; Bollaerts et al., 2017; Hoang et al., 2020), high and/or sustained levels of inflammation can inhibit both injury-induced neurogenesis and axonal regeneration in the CNS (White et al., 2017; Palazzo et al., 2020). As exposure to intra-CNS parasites, and their associated inflammatory responses, can vary dramatically between closely-related organisms that occupy different ecological niches (Scharsack et al., 2007; Peuß et al., 2020), this may account for the loss of regenerative competence in some cold-blooded species.

This model makes several predictions. First, inhibiting regeneration and prolonging reactive gliosis should improve resistance to intra-CNS infection in regeneration-competent species. Second, since this proposed mechanism only leads to a loss of injury-induced neurogenesis, a completely separate molecular mechanism is likely to control loss of injury-induced neurogenesis and axonal regeneration. Third, loss of regenerative competence should directly correlate with increased risk of intra-CNS infection, at least in the recent geological past. This might result from increased parasite load, exposure, and/or pathogenicity.

Testing the first of these predictions is now feasible, using cell-specific, conditional genetic tools and multiomic data available in zebrafish, together with established models of intra-CNS infection (Palha et al., 2013; Passoni et al., 2017; Takaki et al., 2018). Testing the second prediction awaits a thorough analysis of gene regulatory networks controlling the response to axonal injury in both regeneration-competent and incompetent species, as has been done for injury-induced neurogenesis. The third prediction is harder to test, as epidemiological data for wild populations is rarely available and difficult to acquire. However, in some cases, such as surface- and cave-dwelling tetra fish, clear differences in both the parasite load and the response to infection have been described (Peuß et al., 2020), and it should be straightforward to test whether these correlate with differences in regenerative competence.

## What Do We Still Need to Know?

To understand how regenerative competence has been lost over the course of evolution, and to distinguish among these competing hypotheses, additional research is urgently needed. Most importantly, we need a more extensive comparative characterization of regenerative competence. This has been systematically investigated in only a small number of fish, amphibians, and reptiles, and even more rarely has this analysis been done across different CNS regions in individual species. This will both aid efforts to correlate individual ecological, behavioral, and physiological traits with the loss or retention of regenerative competence and provide additional model systems for molecular analysis. This comparative analysis needs to be extended to comprehensive characterization of injury-activated gene regulatory networks in both neurons and glia, investigating whether general principles obtained from the analysis of zebrafish and mice extend more broadly to regeneration-competent and -incompetent organisms, and also determine whether they extend to more broadly regeneration-competent and -incompetent CNS regions within the same species. Finally, given the tantalizing evidence for a link between loss of regenerative competence and resistance to infection, we urgently need a more extensive understanding of how this correlates with overall exposure to pathogens that target the CNS and the resulting immune response, as well as to understand how regeneration-competent organisms respond to these infections (Godwin et al., 2017).

## Author Contributions

SB conceived and wrote this manuscript.

## Conflict of Interest

The author declares that the research was conducted in the absence of any commercial or financial relationships that could be construed as a potential conflict of interest.

## Publisher’s Note

All claims expressed in this article are solely those of the authors and do not necessarily represent those of their affiliated organizations, or those of the publisher, the editors and the reviewers. Any product that may be evaluated in this article, or claim that may be made by its manufacturer, is not guaranteed or endorsed by the publisher.
